# The Rationale for Using Neridronate in Musculoskeletal Disorders: From Metabolic Bone Diseases to Musculoskeletal Pain

**DOI:** 10.3390/ijms23136921

**Published:** 2022-06-22

**Authors:** Giovanni Iolascon, Antimo Moretti

**Affiliations:** Department of Medical and Surgical Specialties and Dentistry, University of Campania “Luigi Vanvitelli”, 81100 Naples, Italy; giovanni.iolascon@unicampania.it

**Keywords:** diphosphonates, 6-amino-1-hydroxyhexane-1,1-diphosphonate, osteogenesis imperfecta, osteoporosis, Paget disease of bone, complex regional pain syndromes, algodystrophy

## Abstract

Neridronate or ((6-amino-1-hydroxy-1-phosphonohexyl) phosphonic acid) is an amino-bisphosphonate (BP) synthetized in Italy in 1986. Bisphosphonates are molecules with a P-C-P bond in their structure that allows strong and selectively binding to hydroxyapatite (HAP) as well as osteoclasts inhibition through different mechanisms of action. Neridronate was initially used to treat Paget disease of the bone, demonstrating effectiveness in reducing bone turnover markers as well as pain. The interesting molecular properties of neridronate foster its wide use in several other conditions, such as osteogenesis imperfecta, and osteoporosis. Thanks to the unique safety and efficacy profile, neridronate has been used in secondary osteoporosis due to genetic, rheumatic, and oncological diseases, including in pediatric patients. In the last decade, this drug has also been studied in chronic musculoskeletal pain conditions, such as algodystrophy, demonstrating effectiveness in improving extraskeletal outcomes. This review highlights historical and clinical insights about the use of neridronate for metabolic bone disorders and musculoskeletal pain conditions.

## 1. Preliminary Remarks

Neridronate is a second-generation bisphosphonate (BP) used for several bone metabolic disorders because of its pharmacokinetics and mechanisms of action [1]. Bisphosphonates are chemically stable analogs of phosphates and pyrophosphates [2]. The latter play primary roles in several biological pathways essential for life. Phosphorus is the eleventh most abundant element in the Earth’s crust, and it exists in living organisms as monovalent or divalent phosphate groups [3]. In the human body, the largest deposit of phosphorus is the skeleton, where 85% of this element is bound to calcium in the form of hydroxyapatite (HAP) [4]. Outside the skeleton, almost all the phosphorus is intracellular, while the extracellular 1% is mostly incorporated in organic macromolecules [5].

Organic phosphate is a key component of a variety of biochemical compounds (i.e., proteins, nucleic acids, and phospholipids) that are involved in several biological processes [6]. Intracellular phosphate is part of information storage (DNA and RNA), signaling pathways, plays a role in the activation of enzymes/proteins through phosphorylation and cyclic adenosine monophosphate (cAMP), and during energy metabolism and the generation of adenosine triphosphate (ATP). This latter consists of two highly reactive bonds that represent a fundamental source of energy to carry out the mechanical, chemical, electrical, thermal, and osmotic work necessary for every living organism [7,8]. In the presence of phosphatase, ATP can transfer onto molecules of other compounds a particular group of its molecule together with a part of the energy associated with it [8]. A very small amount (i.e., about 0.3%) is inorganic phosphate which is commonly measured in biochemical investigations for calcium and phosphorus metabolism [9].

## 2. History of Bisphosphonates

In the 1960s, Herbert Fleisch, studying the factors determining the selective calcification of bone collagen compared to that present in other organs, hypothesized that inorganic polyphosphates, present in body fluids, could be responsible for inhibiting their deposition on extraskeletal collagen [10]. Polyphosphates have been used since the 1940s as calcification inhibitors to prevent encrustations in water systems [11]. Subsequently, it was demonstrated that the physicochemical action of pyrophosphate consisted not only of the inhibition of the crystallization of calcium phosphate from the solution, but also of the inhibition of the aggregation process of calcium phosphate crystals in larger clusters, and finally of the inhibition of the dissolution of HAP crystals [10]. Therefore, the pyrophosphate would protect soft tissues from mineralization, whereas it would affect the calcification rate as well as the dissolution of the preformed mineral in bone. The regulation of its local concentration is carried out by enzymes with pyrophosphatase activity, such as lysosomal alkaline phosphatase (ALP) and acid phosphatase [12,13]. In vivo, pyrophosphate hydrolysis is very rapid; therefore, it was necessary to find analogs of pyrophosphate with similar actions but resistant to enzymatic degradation [14]. However, these compounds, now referred to as BPs, had already been discovered in 1894 by the pharmacist Theodor Salzer, for use in some industrial processes (i.e., textile, fertilizer, and oil industry) as corrosion inhibitors or as complexing agents [15]. Bisphosphonates are characterized by a phosphorus-carbon-phosphorus (P-C-P) bond in the molecular structure that facilitates strong and selective binding to HAP as well as osteoclasts inhibition through distinct mechanisms of action [16]. The first-generation BPs (i.e., non-nitrogen-containing BPs) are incorporated into adenosine monophosphate (AMP), thereby resulting in toxic and non-hydrolyzable analogs of ATP, while second-generation BPs (nitrogen-containing BPs), in addition to the formation of ATP analogs, inhibit enzymes that synthesize key metabolites for osteoclast function and survival [17]. However, phosphorus compounds, such as phosphonates and phosphinates, which are characterized by the presence of highly stable carbon-phosphorus (C-P) bonds instead of labile carbon-oxygen-phosphorus (C-O-P) bonds, are also present in some biomolecules containing phosphorus [18]. Indeed, it has also been found that natural BPs, such as ciliatin or 2-aminoethylphosphonic acid (2-EPA), which are analogs of the amino acids β-alanine and aminosulfonate taurine, are quite common among different organisms, from prokaryotes to eubacteria, mushrooms, mollusks, insects, and others [19,20]. Furthermore, phosphonates form about 10% of the dissolved and particulate phosphorus in the oceans and appear to be key determinants of the productivity of marine phytoplankton [21]. These molecules most likely appear in the form of polysaccharides esterified with methylphosphonic acid and 2-hydroxyethylphosphonic acid [22]. 

## 3. Bisphosphonates as Therapeutic Molecules

The use of BPs as drugs dates back to 1966, stemming from a collaboration between the group of Swiss researchers led by Herbert Fleisch and the research group of Procter and Gamble (P&G) Co. of Cincinnati [23]. P&G demonstrates a long-standing interest in the field of dental care, with the aim of discovering analogs of pyrophosphate to develop a topical agent for use against dental plaque [24,25]. 

Fleisch conducted a series of experiments on BPs synthesized by P&G, which showed both an inhibitory effect in vivo on ectopic calcification in the aortic tissue of rats with hypervitaminosis D and blocking of the dissolution of HAP crystals, suggesting that BPs could in some way delay bone resorption, as confirmed later in various experimental models, including in vivo models [26,27,28].

The first clinical application of BPs occurred in 1967 on a 16-month-old girl with progressive myositis ossifying (MOP) for which Fleisch suggested the use of etidronate to block the progression of calcification [29]. Given the particularly rapid worsening of the disease, the U.S. Food and Drug Administration (FDA) quickly approved the trial and the girl was treated with etidronate orally at a dose of 10 mg/kg per day, leading to the significant regression of the disease starting from the third day of treatment.

On the other side, based on the proven ability of etidronate to inhibit bone resorption, it was hypothesized that it was also effective in high turnover bone diseases such as Paget’s disease of bone (PDB), which was confirmed by a clinical study on humans in which etidronate proved to be effective in reducing some biochemical indexes of bone turnover including serum ALP and urinary hydroxyproline [30]. Subsequent studies confirmed this specific antiresorptive activity; therefore, etidronate was approved for the treatment of PDB, as well as for the prevention and treatment of heterotopic ossification after hip arthroplasty or spinal cord injury, and for malignant hypercalcemia [31,32,33,34,35,36]. 

At the same time, the use of some BPs such as methylene-hydroxy-bisphosphonate, linked to technetium-99 and administered parenterally, for bone scans, was conceived and developed, since this molecular complex can detect abnormally increased bone turnover as in PDB and in bone metastases [37,38].

Pharmacological research led to the synthesis, in the following years, of various molecules belonging to the BP class, which differed in the development of their capacity for pharmacological action and clinical application [2].

Without any doubt, however, osteoporosis is the pathology that has made this class of drugs successful, especially with the synthesis of nitrogen-containing BPs such as alendronate, risedronate, and zoledronate, which when administered significantly reduce the risk of fragility fractures (i.e., vertebral, and non-vertebral fractures) by up to 40% [2].

The potency of BPs as antiresorptive drugs depends on the following two factors: their affinity for HAP and their ability to inhibit osteoclasts [39]. Both factors depend on the molecular properties (e.g., structure and composition) of BPs. First-generation BPs (not containing nitrogen, e.g., etidronate and clodronate) are incorporated into non-hydrolyzable analogs of ATP, which interfere with ATP-dependent intracellular pathways [40]. Second-generation BPs containing nitrogen (e.g., alendronate, risedronate, zoledronate, and neridronate) can interact with key enzymes, such as farnesyl diphosphate synthase (FDPS), of the mevalonate/cholesterol biosynthetic pathway, binding and inhibiting the active site via their N atom [41]. Blocking this pathway prevents the synthesis of isoprenoid compounds which are essential for the post-translational prenylation of small Guanosine-5′-triphosphate (GTP)-binding proteins such as rab, ras, rho, and rac, which are critical for intracellular signaling events in osteoclasts resulting in the inhibition of bone resorption [42,43,44,45].

In recent years, several experimental and clinical studies have been published on the use of these drugs in extraskeletal conditions [46,47]. From a clinical point of view, the studies concerning the use of BPs in the treatment of some protozoal infections [48], in the modulation of the immune response [49], in the mechanisms of tissue repair [50], and generally in improving cell survival [51], could have a significant impact on the management of many diseases. Additionally, in the field of bone health, new research addressed the role of BPs as transporters of other pharmacological substances that would otherwise reach the bone tissue with difficulty and low doses [52,53,54,55]. 

## 4. Neridronate for Treatment of Bone Diseases

### 4.1. Paget Disease of Bone

Neridronic Acid (or neridronate or (6-amino-1-hydroxy-1-phosphonohexyl) phosphonic acid) has the formula C6H17NO7P2 and a molecular weight of 277.15 [56]. This compound was synthetized by Gentili S.p.A. (Pisa, Italy) in 1986 along with a series of other new BPs to investigate their effects on metaphyseal bone remodeling, mineralization, density, their trabecular number, and diameter in rats [57]. In the study conducted by Schenk et al. only alendronate and neridronate were deemed to be interesting for future clinical use according to the strength of inhibition of bone resorption and the limited inhibition of bone formation along with a lower cellular toxicity compared to other BPs (i.e., 5-amino-1-hydroxypentylidene-1,1–bisphosphonate, 3-amino- 1-hydroxypropylidene- 1,1-bisphosphonate, and dichloromethylene bisphosphonate). Indeed, in the following year, Atkins et al. [58]. investigated the effects of neridronate in 42 patients with PDB, demonstrating that its oral (400 mg daily for 1 month) or IV (25 or 50 mg daily for 5 days) administration significantly suppressed biochemical markers of disease activity, such as urinary hydroxyproline (up to −42% at the end of treatment) and serum ALP (up to −49% at 3-month follow-up) with the maintenance of these results over 6 months and no adverse effects on mineralization at both Pagetic sites and uninvolved bone. Moreover, all but three patients reported bone pain relief after neridronate therapy. 

### 4.2. Osteogenesis Imperfecta

The high expectations of the efficacy and safety of the drug led years later to the second indication for clinical use of neridronate (i.e., osteogenesis imperfecta, OI), driven by two Italian randomized controlled trials (RCTs) on behalf of the Italian Ministry of Health and the Italian Association of patients with OI (AsItOI) [59,60]. 

In the first study, Adami et al. [59] randomized 46 adults (23 men and 23 premenopausal women) with OI to receive IV neridronate (100 mg every 3 months) or no intervention, demonstrating progressive improvements in both spine and hip BMD at 1- (+3% and +4.3%, respectively) and 2-year (additional increases of 3.9% and 1.5%, respectively) follow-ups in the neridronate group, along with a significantly lower fracture incidence compared to controls. Moreover, significant reductions in bone turnover markers (BTMs), such as serum bone alkaline phosphatase (BAP; −20%), serum carboxy-terminal collagen crosslinks (CTX; −25%), and urinary free-deoxy pyridinoline (ufDPD; −20%) were reported in patients receiving neridronate 3 months after the last infusion. Concerning safety, 13 patients reported flu-like symptoms 1–2 days after the first IV administration, which lasted a few hours. 

In the second study, Gatti et al. [60] investigated the efficacy of IV neridronate (2 mg/kg every 3 months) in terms of bone density improvement and fracture risk reduction in prepubertal children with OI, demonstrating significant increases in both the spine and hip BMD of 18–25% and reduced incidence of fragility fractures (−64%) in those receiving the intervention compared to the control group (no treatment) at 1-year follow-up. Surprisingly, both total ALP and BALP progressively declined in the intervention group compared to controls at 1-year, considering that in postmenopausal osteoporosis these changes tend to stabilize after 6 months of neridronate treatment. However, the authors claimed that the implications of this finding remain uncertain. This RCT confirmed the good safety profile of neridronate reported in a similar study performed on adults with OI. Furthermore, in a study including OI infants receiving IV neridronate (2 mg/kg for 2 consecutive days, every 3 months) from the neonatal period, significant improvements in growth parameters (weight and length) were reported compared to both infants of 6 months of age receiving the same treatment and to untreated OI infants [61].

More than 10 years later, long-term data about the effectiveness and safety of neridronate in both children and adults with OI were reported. Idolazzi et al. [62] administered IV neridronate at 2 mg/kg (maximum dose 100 mg) every 3 months for 3 years to 55 young patients (mean age 12.6 years), reporting statistically significant progressive BMD improvements at both lumbar spine and total hip, despite no effect on fracture-risk reduction, even if the authors reported a significantly lower number of incident fractures in patients with fragility fractures in the 3-year period before the study. Moreover, no negative effects on skeletal growth were reported, as suggested by the significantly increased bone area. In the same year, Viapiana et al. [63] published an observational 3-year study investigating the effectiveness and safety of the same dosing regimen of neridronate on the same outcomes in adults, reporting similar benefits in terms of BMD changes, and confirming an insignificant fracture risk reduction.

### 4.3. Primary Osteoporosis

In 2003, the first paper on the use of neridronate for the treatment of osteoporosis was published [64]. The clinical trial of Braga et al. investigated the efficacy and safety of IV neridronate (50 mg bimonthly for 2 years) and calcium and vitamin D supplementation on postmenopausal women with osteoporosis (spine BMD T-score < −2.5 SD) compared to calcium and vitamin D supplementation. The intervention group reported significant BMD improvements at both spine and femoral neck (+7.4% and +5.8%, respectively) at 2-year follow-up, which were maintained 1 year later. These findings were accompanied by significant reductions in BTM within 2 months, reaching a plateau at 6 months (−35% for BALP) and 2 years (−47% for serum CTX), thus returning to baseline values after 1 year from the end of neridronate therapy. The densitometric changes at lumbar spine and at femoral neck induced by neridronate at 1-year follow-up (about +6 and +4%, respectively) were comparable to those reported after 1 year of oral alendronate [65] (about +5 and + 3%) as well as other parenteral BPs, including zoledronate (about +4.3–5.1% and +3.1–3.5%) [66] and ibandronate (+5.2% and +2.9%) [67], despite biochemical changes of BTM being lower than those observed with alendronate [68] or risedronate [69]. Two years later, Cascella et al. [70] also confirmed the benefits of this drug with the IM route of administration (25 mg monthly) combined with calcium and vitamin D supplementation in a randomized pilot study. The authors investigated the efficacy of this intervention on BMD, BTM, and quality of life (QoL) in postmenopausal women with osteoporosis. After 1-year of therapy, the intervention group reported significant changes in the spine and hip BMD (+6.6% and +4.2%, respectively), along with significant BTM reductions (−34.2% for BALP, −38% for serum CTX, −25.2% for ufDPD) starting at 3 months of treatment, compared to women receiving only calcium and vitamin D supplementation. Moreover, significant improvements in QoL were also reported, particularly for bodily pain, general health perception, vitality, emotional role functioning, and physical role functioning. In 2008, Adami et al. [71] confirmed significant densitometric and biochemical changes obtained with IM neridronate (25 mg every 2 weeks, 12.5 or 25 mg every 4 weeks) in post-menopausal women with osteoporosis in a phase 2 clinical trial compared to placebo, also providing a 2-year follow-up. The authors reported a dose–response relationship in the different dosing regimens for the BMD changes at the total hip but not at the spine, with values significantly higher than the baseline for only the 50 mg monthly dose at a 2-year follow-up.

### 4.4. Secondary Osteoporosis

Some years later, the safety and efficacy of neridronate on secondary osteoporosis were also investigated. In 2011, Rossini et al. [72] studied the long-term effects of this drug (100 mg IV every 2 months for 2 years) in 60 postmenopausal women with primary hyperparathyroidism (PHPT), reporting progressive BMD improvements at both spine (+6.7%) and femoral neck (+2.9%) at the end of the intervention as well as maintenance of BMD gains at the lumbar spine 2 years from discontinuation. Moreover, serum CTX significantly decreased at 6 months and returned to baseline values after 6 months from neridronate discontinuation. On the other hand, serum PTH significantly increased during the treatment period and persisted at a higher level during discontinuation, although this finding was reported in patients with vitamin D deficiency.

This drug has also been used in rheumatic conditions with surprising results in terms of ancillary effects, particularly anti-inflammatory properties. Mazzantini et al. [73] administered single IV neridronate at 25 mg and 50 mg compared to placebo in patients with active rheumatoid arthritis (RA), reporting statistically significant reductions in the erythrocyte sedimentation rate (ESR) and C-reactive protein (CRP) only in the group treated with 25 mg neridronate compared to patients treated with higher doses of the active drug and to those receiving placebo, respectively, along with significant reductions in N-telopeptide of type I collagen (NTx) and hydroxyproline (OHP) in the intervention groups at 1 week. Many years later, monthly IV neridronate (100 mg) was compared to infliximab (mg/kg) to investigate the anti-inflammatory and bone metabolic effects of these interventions in patients with ankylosing spondylitis (AS) [74]. In both groups, comparable benefits in terms of reduced disease activity (i.e., Bath Ankylosing Spondylitis Disease Activity Index, BASDAI, and Bath Ankylosing Spondylitis Functional Index, BASFI), as well as a significant increase in BMD in the neridronate group, were only reported in the short term (3 to 6 months).

The efficacy of this drug has been studied also in cancer patients, particularly in malignant hypercalcemia, in men receiving androgen deprivation therapy (ADT), and in patients with multiple myeloma (MM). In 1994, neridronate (single IV infusion of 125 mg) was used for the first time to manage hypercalcemia due to malignancy in 20 patients [75], demonstrating a serum calcium fall within 3 days that remained low for up to 28 days, with 65% of patients reporting normocalcemia, without adverse events. 

In 2004, the first study including advanced prostate cancer patients treated with bicalutamide was conducted to investigate the efficacy of IM neridronate (25 mg monthly), calcium (500 mg) and vitamin D (400 IU) compared to calcium and vitamin D supplementation alone at 1-year follow-up [76]. The authors demonstrated that the intervention group reported no significant changes in deoxypyridinoline (DPD) and BALP as well as an unchanged lumbar spine and total hip BMD compared to the other group, suggesting a role of neridronate in preventing bone loss in this population. These findings were confirmed by Magno et al. [77] in a similar study published the following year, reporting comparable results in terms of BTM and BMD changes at 1-year follow-up in patients treated with ADT for prostate cancer receiving the same dosage of neridronate. Along the lines of these encouraging results, Pittari et al. [78] investigated the efficacy of this drug (monthly IV administration of 100 mg) in a small sample (7 patients) of MM patients with pathologic fractures or bone loss, reporting spine BMD improvement without adverse events at 1-year follow-up.

More recently, Giannini et al. [79] carried out a multicenter randomized placebo-controlled trial to investigate the efficacy of IM neridronate (25 mg monthly) in patients with bone loss after a solid organ transplant (i.e., heart, lung, or liver transplantation), reporting a statistically significant increase in lumbar spine BMD at 12 months in the intervention group along with no between-group difference in the safety profile. 

The effectiveness of neridronate has been suggested also in patients with Duchenne Muscular Dystrophy (DMD) receiving glucocorticoids (GCs). In this population, 1-year treatment with IM neridronate (25 mg monthly), seems to counteract the BMD loss and to prevent incident fragility fractures [80].

In a randomized, open-label study, Forni et al. [81] included adults with β-thalassaemia with low bone density (BMD Z-score < −2 SD) treated with IV neridronate (100 mg every 3 months) combined with calcium and vitamin D supplementation, reporting significant improvements in the spine and total hip BMD at both 6 and 12-month follow-ups compared to control group (calcium and vitamin D supplementation) along with significant reductions of BALP and serum CTX within the first 3 months. Neridronate was also safe in this population, considering that only 3 out of 54 patients reported flu-like symptoms, which were well-controlled by acetaminophen. Furthermore, interesting findings emerged in this trial of early changes in musculoskeletal pain, a common concern in patients with β-thalassaemia. After 3 months of therapy (only one IV neridronate administration), patients reported significant back pain relief and reduced analgesic use. 

## 5. Neridronate for Treatment of Chronic Musculoskeletal Pain

In the wake of the ancillary effects of neridronate on pain relief, growing evidence supports the role of this drug in musculoskeletal conditions, such as Complex Regional Pain Syndrome (CRPS) type 1 (or algodystrophy) [82,83]. In 2013, the first RCT investigating the efficacy and safety of this drug for the management of musculoskeletal pain as the primary endpoint was published, leading to its approval for the treatment of the CRPS type 1 by a national regulatory agency (The Italian Medicines Agency, AIFA) [84]. Varenna et al. demonstrated that IV neridronate (100 mg four times over 10 days) was significantly more effective than placebo in improving pain (73.2% of patients reported >50% VAS score reduction) at 40-day follow-up. Moreover, in the open extension phase, the placebo group received the same therapeutic regimen as the control group, reporting similar benefits. At 1 year, no patients reported CRPS type 1 symptoms. These results were accompanied by evidence of its efficacy in terms of other clinical (resolution of edema, pain at passive motion, allodynia, hyperalgesia, and better QoL) and instrumental (i.e., bone edema reduction and bone scan uptake normalization) findings of algodystrophy. More recently, comparable results were obtained following the IM neridronate administration of the equivalent dose (25 mg daily for 16 days) in CRPS type 1 patients [85]. In this RCT, clinically relevant benefits were reported in as early as a 30-day follow-up. In both RCTs, no serious adverse events were reported.

**Figure 1:**
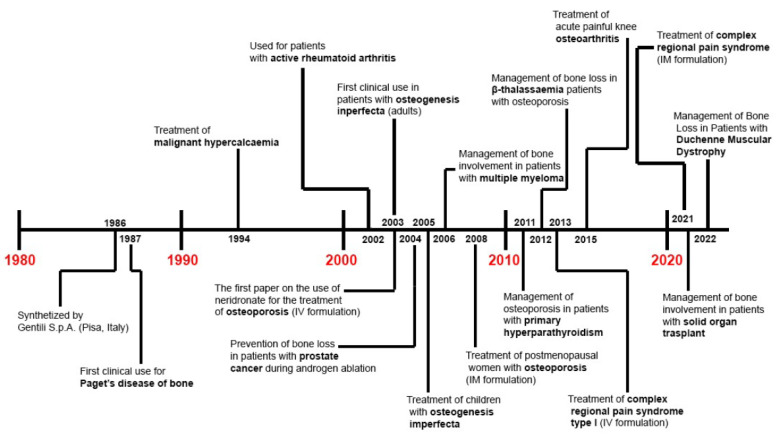
Roadmap of clinical use of neridronate.

Finally, the efficacy of IV neridronate (the same treatment protocol as for CRPS type 1) has been also evaluated for acute pain relief in patients with knee osteoarthritis (KOA) and bone marrow lesions (BMLs) versus placebo [86]. Varenna et al. reported statistically significant pain relief at the end of the intervention in their neridronate group (−48.4%) that further improved 50 days later (−69.1%). Additionally, a statistically significant reduction in BML (i.e., whole-organ MRI score, WORMS, −41.1% in neridronate group) and pain killers use (12.9% vs. 72%) was reported in the intervention group versus placebo (in Figure 1 and Table 1 the history of the clinical use and main findings from clinical studies about neridronate are provided). 

## 6. Conclusions

Since its discovery almost 40 years ago, neridronate has undergone different phases of clinical development, starting from its use in rare diseases, such as PDB and OI, then for the treatment of patients with primary osteoporosis or bone loss secondary to RA and AS, MM, treatments for prostate cancer, PHPT, DMD, and β-thalassemia. Beyond its interesting molecular properties, which place neridronate in an intermediate position among the nitrogen-containing BPs in terms of its HAP binding and osteoclasts’ inhibition, findings drawn from clinical investigations in several conditions suggest that this drug has a unique balance of safety and an efficacy profile that makes it a suitable therapeutic agent not only for metabolic bone disorders but also for musculoskeletal pain conditions such as algodystrophy. Moreover, the efficacy of neridronate has been also studied in patients with KOA, opening new pathways for a molecule originally developed for its anti-resorptive properties, but which over time has shown interesting ancillary effects that could suggest its use for musculoskeletal pain. Findings drawn from clinical investigations in its long history suggest that we have not yet fully understood the potential of neridronate, and future basic research and clinical trials might clarify its range of applicability.

The chemical structure and the consequent pharmacodynamic and pharmacokinetic characteristics underlie the potency of neridronate and its tolerability similarly to other BPs. Evidence of its use in MBD in both pediatric and adult populations makes it an ideal BP for the management of diseases such as OI and PDB. Furthermore, recent evidence demonstrates its efficacy and safety also in chronic painful regional diseases is higher than other BPs. Particularly in algodystrophy, neridronate was effective in modulating pathogenic mechanisms of the disease resulting in more convincing clinical findings compared to other BPs due to the study design, sample size, endpoints and length of follow up of the available trials.

## Figures and Tables

**Table 1:** Main findings from the studies investigating efficacy/effectiveness of neridronate in different clinical conditions.

Authors	Study Design	Clinical Condition	Administration Route	Sample Size	Main Findings
Atkins et al. [58]	Nonrandomized trial	Paget’s disease of bone	Oral (400 mg daily for 1 month) or IV (25–50 mg daily for 5 days)	37	Reduction in BTM (−42% hydroxyproline, −49% BAP). Pain relief in all except 3 patients.
Adami et al. [59]	RCT	OI	IV (100 mg every 3 months)	46	Progressive spine and hip BMD improvements at 1- and 2-year follow-ups. Reduction in BTMs (BALP, serum CTX, ufDPD).
Gatti et al. [60]	RCT	OI	IV (2 mg/kg every 3 months)	64	Spine and hip BMD improvements and reduced fracture risk (−64%).
Antoniazzi et al. [61]	Nonrandomized study	OI	IV (2 mg/kg for 2 consecutive days, every 3 months)	20	Improvements in growth parameters (weight and length) and lower fracture incidence in neonates receiving neridronate vs. both infants of 6 months receiving the same treatment and untreated OI infants.
Idolazzi et al. [62]	Open label, not controlled study	OI	IV (2 mg/kg, maximum dose 100 mg) every 3 months for 3 years	55	BMD improvements at both spine (+50.7%) and total hip (+41.4%) and reduced fracture risk in patients with fragility fractures in the 3-year period before the study at 36 months.
Viapiana et al. [63]	Observational	OI	IV (2 mg/kg, maximum dose 100 mg) every 3 months for 3 years	114	BMD improvements at both spine and total hip without significant reduction in fracture incidence.
Braga et al. [64]	RCT	PMO	IV (50 mg bimonthly for 2 years)	78	Spine and femoral neck BMD improvements. Reductions in BTM (BALP, serum CTX).
Cascella et al. [70]	Randomized pilot study	PMO	IM (25 mg monthly)	40	Spine and hip BMD improvements. BTM reductions (BALP, serum CTX, ufDPD). QoL improvements.
Adami et al. [71]	RCT	PMO	IM (25 mg every 2 weeks, 12.5 or 25 mg every 4 weeks)	188	Dose–response relationship for BMD changes of the total hip.
Mazzantini et al. [73]	Nonrandomized trial	RA	IV (single dose of 25 or 50 mg)	45	Reductions in ESR, CRP, NTx, OHP at 1-week follow-up.
Viapiana et al. [74]	Open label study	AS	IV (100 mg monthly)	60	Benefits in terms of reduced disease activity comparable to infliximab. BMD improvements in the short term (3–6 months).
Rossini et al. [72]	Nonrandomized trial	PHPT	IV (100 mg every 2 months for 2 years)	60	BMD improvements at both spine (+6.7%) and femoral neck (+2.9%) at the end of the intervention and maintenance of BMD gains at lumbar spine after 2 years from discontinuation. Decreased CTX at 6 months and return to baseline values after 6 months from neridronate discontinuation. Increased serum PTH during treatment period in patients with vitamin D deficiency.
Forni et al. [81]	Randomized open-label study	β-thalassaemia with low bone density (BMD Z-score < −2 SD) patients	IV (100 mg every 3 months)	118	Spine and total hip BMD improvements. Back pain relief.
O’Rourke et al. [75]	Pilot study	Malignant hypercalcemia	IV (single dose of 125 mg)	20	Serum calcium fall within 3 days. Normocalcemia in 65% of cases.
Morabito et al. [76]	RCT	ADT	IM (25 mg monthly)	48	No significant changes in DPD, BALP, spine and total hip BMD at 1-year follow-up.
Magno et al. [77]	RCT	ADT	IM (25 mg monthly)	60	No significant changes in DPD, BALP, spine and total hip BMD at 1-year follow-up.
Pittari et al. [78]	Nonrandomized trial	MM	IV (100 mg monthly)	7	Spine BMD improvement (+3.5%) without adverse events at 1-year follow-up.
Giannini et al. [79]	RCT	Solid organ transplant	IM (25 mg monthly)	39	Spine BMD improvement (+0.92 g/cm^2^) at 1-year follow-up.
Moretti et al. [80]	Retrospective pilot study	Duchenne Muscular Dystrophy patients receiving glucocorticoids	IM (25 mg every month) for 1 year	8	Spine BMD maintenance. No incident fragility fractures.
Varenna et al. [84]	RCT	CRPS type 1	IV (100 mg four times over 10 days)	82	Pain relief in 73.2% of patients.
Varenna et al. [85]	RCT	CRPS type 1	IM (25 mg daily for 16 days)	78	Pain relief in 65.9% of patients.
Varenna et al. [86]	RCT	KOA	IV (100 mg four times over 10 days)	60	Pain relief at the end of treatment (−48.4%) and at 50 day-follow-up (−69.1%). Reductions in both bone edema and analgesic drugs use.

Abbreviations: ADT: androgen deprivation therapy; AS: ankylosing spondylitis; BALP: bone alkaline phosphatase; BMD: bone mineral density; BTM: bone turnover markers; CRP: C-reactive protein; CTX: carboxy-terminal collagen crosslinks; CRPS: Complex Regional Pain Syndrome; DPD: deoxypyridinoline; ESR: erythrocyte sedimentation rate; IM: intramuscular; IV: intravenous; KOA: knee osteoarthritis; MM: multiple myeloma; NTx: N-telopeptide of type I collagen; OHP: hydroxyproline; OI: osteogenesis imperfecta; PHPT: primary hyperparathyroidism; PMO: post-menopausal osteoporosis; RA: rheumatoid arthritis; RCT: randomized controlled trial; ufDPD: urinary free-deoxy pyridinoline.

## Data Availability

Not applicable.

## References

[1] Gatti D., Viapiana O., Idolazzi L., Fracassi E., Adami S. (2009). Neridronic acid for the treatment of bone metabolic diseases. Expert Opin. Drug Metab. Toxicol..

[2] Russell R.G.G. (2011). Bisphosphonates: The first 40 years. Bone.

[3] Paytan A., McLaughlin K. (2007). The Oceanic Phosphorus Cycle. Chem. Rev..

[4] Michigami T., Ozono K. (2019). Roles of Phosphate in Skeleton. Front. Endocrinol..

[5] Kritmetapak K., Kumar R. (2021). Phosphate as a Signaling Molecule. Calcif. Tissue Res..

[6] Peacock M. (2021). Phosphate Metabolism in Health and Disease. Calcif. Tissue Res..

[7] De Meis L. (2012). How Enzymes Handle the Energy Derived from the Cleavage of High-energy Phosphate Compounds. J. Biol. Chem..

[8] Ross J. (2006). Energy Transfer from Adenosine Triphosphate. J. Phys. Chem. B.

[9] Iheagwara O.S., Ing T.S., Kjellstrand C.M., Lew S.Q. (2013). Phosphorus, phosphorous, and phosphate. Hemodial. Int..

[10] Fleisch H., Russell R.G.G., Straumann F. (1966). Effect of Pyrophosphate on Hydroxyapatite and Its Implications in Calcium Homeostasis. Nature.

[11] Larson T.E. (1957). Evaluation of the Use of Polyphosphates in the Water Industry. J. Am. Water Work. Assoc..

[12] Moss D.W., Eaton R.H., Smith J.K., Whitby L.G. (1967). Association of inorganic-pyrophosphatase activity with human alkaline-phosphatase preparations. Biochem. J..

[13] Brightwell R., Tappel A. (1968). Lysosomal acid pyrophosphatase and acid phosphatase. Arch. Biochem. Biophys..

[14] Russell R.G. (1976). Metabolism of inorganic pyrophosphate (PPi). Arthritis Rheum..

[15] Petroianu G.A. (2011). Pharmacist Theodor Salzer (1833–1900) and the discovery of bisphosphonates. Pharmazie.

[16] Fleisch H. (1981). Diphosphonates: History and mechanisms of action. Metab. Bone Dis. Relat. Res..

[17] Russell R.G.G., Rogers M.J., Frith J.C., Luckman S.P., Coxon F.P., Benford H.L., Croucher P.I., Shipman C., Fleisch H.A. (1999). The pharmacology of bisphosphonates and new insights into their mechanisms of action. J. Bone Miner. Res..

[18] Metcalf W.W., van der Donk W.A. (2009). Biosynthesis of Phosphonic and Phosphinic Acid Natural Products. Annu. Rev. Biochem..

[19] Alhadeff J.A., Daves G.D. (1971). 2-Aminoethylphosphonic acid: Distribution in human tissues. Biochim. Biophys. Acta..

[20] Quin L.D. (1964). 2-Aminoethylphosphonic Acid in Insoluble Protein of the Sea Anemone *Metridium dianthus*. Science.

[21] Acker M., Hogle S.L., Berube P.M., Hackl T., Coe A., Stepanauskas R., Chisholm S.W., Repeta D.J. (2022). Phosphonate production by marine microbes: Exploring new sources and potential function. Proc. Natl. Acad. Sci. USA.

[22] Kafarski P., Churchill D.G., Sikirić M.D., Čolović B., Milhofer H.F. (2019). Phosphonates: Their Natural Occurrence and Physiological Role. Contemporary Topics about Phosphorus in Biology and Materials.

[23] Fleisch H., Maerki J., Russell R.G.G. (1966). Effect of Pyrophosphate on Dissolution of Hydroxyapatite and Its Possible Importance in Calcium Homeostasis. Proc. Soc. Exp. Bio..

[24] Francis M.D., Briner W.W. (1973). The effect of phosphonates on dental enamel in vitro and calculus formation in vivo. Calcif. Tissue Res..

[25] Francis M.D. (1969). The inhibition of calcium hydroxypatite crystal growth by polyphosphonates and polyphosphates. Calcif. Tissue Res..

[26] Francis M.D., Graham R., Russell G., Fleisch H. (1969). Diphosphonates Inhibit Formation of Calcium Phosphate Crystals in vitro and Pathological Calcification in vivo. Science.

[27] Fleisch H., Graham R., Russell G., Francis M.D. (1969). Diphosphonates Inhibit Hydroxyapatite Dissolution in vitro and Bone Resorption in Tissue Culture and in vivo. Science.

[28] Fleisch H., Russell R.G.G., Simpson B., Muhlbauer R.C. (1969). Prevention by a diphosphonate of immobilisation ‘osteoporosis’ in rats. Nature.

[29] Bassett C., Donath A., Macagno F., Preisig R., Fleisch H., Francis M. (1969). Diphosphonates in the treatment of myositis ossificans. Lancet.

[30] Smith R., Russell R.G.G., Bishop M.C. (1971). Diphosphonates and Paget’s disease of bone. Lancet.

[31] Russell R.G.G., Smith R., Preston C., Walton R.J., Woods C.F. (1974). Diphosphonates in Paget’s disease. Lancet.

[32] Johnston C.C., Khairi M.R., Meunier P.J. (1980). Use of etidronate (EHDP) in Paget’s disease of bone. Arthritis. Rheum..

[33] Bijvoet O.L.M., Nollen A.J.G., Slooff T.J.J.H., Feith R. (1974). Effect of a Diphosphonate on Para-Articular Ossification after total Hip Replacement. Acta Orthop. Scand..

[34] Merli G.J., McElwain G.E., Adler A.G., Martin J.H., Roberts J.D., Schnall B., Ditunno J.F. (1984). Immobilization hypercalcemia in acute spinal cord injury treated with etidronate. Arch. Intern. Med..

[35] Pearson E.G., Nance P.W., Leslie W.D., Ludwig S. (1997). Cyclical etidronate: Its effect on bone density in patients with acute spinal cord injury. Arch. Phys. Med. Rehabil..

[36] Ryzen E., Martodam R.R., Troxell M., Benson A., Paterson A., Shepard K., Hicks R. (1985). Intravenous etidronate in the management of malignant hypercalcemia. Arch. Intern. Med..

[37] Tofe A.J., Francis M.D. (1974). Optimization of the ratio of stannous tin: Ethane-1-hydroxy-1, 1-diphosphonate for bone scanning with 99mTc-pertechnetate. J. Nucl. Med..

[38] Fogelman I., Bessent R.G., Turner J.F., Citrin D.L., Boyce I.T., Greig W.R. (1978). The use of whole-body retention of Tc-99m diphosphonate in the diagnosis of metabolic bone disease. J. Nucl. Med..

[39] Lundy M.W., Ebetino F.H., Fei L., Xia Z., Pozzi M., Trokhan D., Phipps R., Dunford J., Triffitt J., Russell R. (2007). Bisphosphonate affinity to hydroxyapatite and farnesyl pyrophosphate inhibitory potency together drive in vivo efficacy. J. Bone Miner. Res..

[40] Rogers M.J., Crockett J.C., Coxon F.P., Mönkkönen J. (2011). Biochemical and molecular mechanisms of action of bisphosphonates. Bone.

[41] Kavanagh K.L., Guo K., Dunford J.E., Wu X., Knapp S., Ebetino F.H., Rogers M.J., Russell R.G.G., Oppermann U. (2006). The molecular mechanism of nitrogen-containing bisphosphonates as antiosteoporosis drugs. Proc. Natl. Acad. Sci. USA.

[42] Coxon F.P., Rogers M. (2003). The Role of Prenylated Small GTP-Binding Proteins in the Regulation of Osteoclast Function. Calcif. Tissue Int..

[43] Hall A. (1998). Rho GTPases and the Actin Cytoskeleton. Science.

[44] Olkkonen V.M., Slenmark H. (1997). Role of Rab GTPases in Membrane Traffic. Int. Rev. Cytol..

[45] Glomset J.A., Gelb M.H., Farnsworth C.C. (1992). Geranylgeranylated proteins. Biochem. Soc. Trans..

[46] Panagiotakou A., Yavropoulou M., Nasiri-Ansari N., Makras P., Basdra E.K., Papavassiliou A.G., Kassi E.N. (2020). Extra-skeletal effects of bisphosphonates. Metabolism.

[47] Body J.-J., Bergmann P., Boonen S., Devogelaer J.-P., Gielen E., Goemaere S., Kaufman J.-M., Rozenberg S., Reginster J.-Y. (2012). Extraskeletal benefits and risks of calcium, vitamin D and anti-osteoporosis medications. Osteoporos. Int..

[48] Santos J.C.B., De Melo J.A., Maheshwari S., de Medeiros W.M.T.Q., de Freitas Oliveira J.W., Moreno C., Amzel L.M., Gabelli S.B., Silva M.S. (2020). Bisphosphonate-Based Molecules as Potential New Antiparasitic Drugs. Molecules.

[49] George C.N., Canuas-Landero V., Theodoulou E., Muthana M., Wilson C., Ottewell P. (2020). Oestrogen and zoledronic acid driven changes to the bone and immune environments: Potential mechanisms underlying the differential anti-tumour effects of zoledronic acid in pre- and post-menopausal conditions. J. Bone Oncol..

[50] Sharma D., Hamlet S.M., Petcu E.B., Ivanovski S. (2016). The effect of bisphosphonates on the endothelial differentiation of mesenchymal stem cells. Sci. Rep..

[51] Walter C., Pabst A., Ziebart T., Klein M., Al-Nawas B. (2011). Bisphosphonates affect migration ability and cell viability of HUVEC, fibroblasts and osteoblasts in vitro. Oral Dis..

[52] Adjei-Sowah E., Peng Y., Weeks J., Jonason J., Bentley K.D.M., Masters E., Morita Y., Muthukrishnan G., Cherian P., Hu X. (2021). Development of Bisphosphonate-Conjugated Antibiotics to Overcome Pharmacodynamic Limitations of Local Therapy: Initial Results with Carbamate Linked Sitafloxacin and Tedizolid. Antibiotics.

[53] Tal N., Rudnick-Glick S., Grinberg I., Natan M., Banin E., Margel S. (2018). Engineering of a New Bisphosphonate Monomer and Nanoparticles of Narrow Size Distribution for Antibacterial Applications. ACS Omega.

[54] Cabezón E., de la Cruz F., Arechaga I. (2017). Conjugation Inhibitors and Their Potential Use to Prevent Dissemination of Antibiotic Resistance Genes in Bacteria. Front. Microbiol..

[55] Forsgren J., Brohede U., Strømme M., Engqvist H. (2011). Co-loading of bisphosphonates and antibiotics to a biomimetic hydroxyapatite coating. Biotechnol. Lett..

[56] PubChem (2004). Bethesda (MD): National Library of Medicine (US), National Center for Biotechnology Information. PubChem Compound Summary for CID 71237. Neridronic Acid. https://pubchem.ncbi.nlm.nih.gov/compound/Neridronic-acid.

[57] Schenk R., Eggli P., Fleisch H., Rosini S. (1986). Quantitative morphometric evaluation of the inhibitory activity of new aminobisphosphonates on bone resorption in the rat. Calcif. Tissue Res..

[58] Atkins R.M., Yates A.J., Gray R.E., Urwin G.H., Hamdy N.A., Beneton M.N., Rosini S., Kanis J.A. (1987). Aminohexane diphosphonate in the treatment of paget’s disease of bone. J. Bone Miner. Res..

[59] Adami S., Gatti D., Colapietro F., Fracassi E., Braga V., Rossini M., Tatò L. (2003). Intravenous Neridronate in Adults With Osteogenesis Imperfecta. J. Bone Miner. Res..

[60] Gatti D., Antoniazzi F., Prizzi R., Braga V., Rossini M., Tatò L., Viapiana O., Adami S. (2005). Intravenous Neridronate in Children with Osteogenesis Imperfecta: A Randomized Controlled Study. J. Bone Miner. Res..

[61] Antoniazzi F., Zamboni G., Lauriola S., Donadi L., Adami S., Tatò L. (2006). Early bisphosphonate treatment in infants with severe osteogenesis imperfecta. J. Pediatr..

[62] Idolazzi L., Fassio A., Viapiana O., Rossini M., Adami G., Bertoldo F., Antoniazzi F., Gatti D. (2017). Treatment with neridronate in children and adolescents with osteogenesis imperfecta: Data from open-label, not controlled, three-year Italian study. Bone.

[63] Viapiana O., Idolazzi L., Fassio A., Orsolini G., Rossini M., Adami G., Bertoldo F., Gatti D. (2017). Long-term Effects of Neridronate in Adults with Osteogenesis Imperfecta: An Observational Three-Year Italian Study. Calcif. Tissue Res..

[64] Braga V., Gatti D., Colapietro F., Battaglia E., Righetti D., Prizzi R., Rossini M., Adami S. (2003). Intravenous intermittent neridronate in the treatment of postmenopausal osteoporosis. Bone.

[65] Liberman U.A., Weiss S.R., Broll J., Minne H.W., Quan H., Bell N.H., Rodriguez-Portales J., Downs R.W., Dequeker J., Favus M. (1995). Effect of oral alendronate on bone mineral density and the incidence of fractures in postmenopausal osteoporosis. N. Engl. J. Med..

[66] Reid I.R., Brown J.P., Burckhardt P., Horowitz Z., Richardson P., Trechsel U., Widmer A., Devogelaer J.P., Kaufman J.M., Jaeger P. (2002). Intravenous zoledronic acid in postmenopausal women with low bone mineral density. N. Engl. J. Med..

[67] Thiebaud D., Burckardt P., Kriegbaum H., Huss H., Mulder H., Juttmann J.R., Schoter K.H. (1997). Three monthly intravenous injections of ibandronate in the treatment of postmenopausal osteoporosis. Am. J. Med..

[68] Chesnut C.H., McClung M.R., Ensrud K.E., Bell N.H., Genant H.K., Harris S.T., Singer S.R., Stock J.L., Yood R.A., Delmas P.D. (1995). Alendronate treatment of the postmenopausal osteoporotic woman: Effect of multiple dosages on bone mass and bone remodeling. Am. J. Med..

[69] Harris S.T., Watts N.B., Genant H.K., McKeever C.D., Hangartner T., Keller M., Chesnut C.H., Brown J., Eriksen E.F., Hoseyni M.S. (1999). Effects of risedronate treatment on vertebral and non-vertebral fractures in women with postmenopausal osteoporosis. A randomized controlled trial. J. Am. Med. Assoc..

[70] Cascella T., Musella T., Orio F., Palomba S., Bifulco G., Nappi C., Lombardi G., Colao A., Tauchmanova L. (2005). Effects of neridronate treatment in elderly women with osteoporosis. J. Endocrinol. Investig..

[71] Adami S., Gatti D., Bertoldo F., Sartori L., Di Munno O., Filipponi P., Marcocci C., Frediani B., Palummeri E., Fiore C.E. (2008). Intramuscular Neridronate in Postmenopausal Women with Low Bone Mineral Density. Calcif. Tissue Res..

[72] Rossini M., Viapiana O., Kalpakcioglu B., Dhangana R., Gatti D., Braga V., Fracassi E., Adami S. (2011). Long-Term Effects of Neridronate and its Discontinuation in Patients with Primary Hyperparathyroidism. Calcif. Tissue Res..

[73] Mazzantini M., Di Munno O., Metelli M.R., Bulleri M., Giordani R. (2002). Single infusion of neridronate (6-amino-1-hydroxyhexylidene-1,1-bisphosphonate) in patients with active rheumatoid arthritis: Effects on disease activity and bone resorption markers. Aging Clin. Exp. Res..

[74] Viapiana O., Gatti D., Idolazzi L., Fracassi E., Adami S., Troplini S., Povino M.R., Rossini M. (2014). Bisphosphonates vs infliximab in ankylosing spondylitis treatment. Rheumatology.

[75] O’Rourke N., McCloskey E., Rosini S., Coleman R., Kanis J. (1994). Treatment of malignant hypercalcaemia with aminohexane bisphosphonate (neridronate). Br. J. Cancer.

[76] Morabito N., Gaudio A., Lasco A., Catalano A., Atteritano M., Trifiletti A., Anastasi G., Melloni D., Frisina N. (2004). Neridronate Prevents Bone Loss in Patients Receiving Androgen Deprivation Therapy for Prostate Cancer. J. Bone Miner. Res..

[77] Magno C., Anastasi G., Morabito N., Gaudio A., Maisano D., Franchina F., Galì A., Frisina N., Melloni D. (2005). Preventing Bone Loss During Androgen Deprivation Therapy for Prostate Cancer: Early Experience with Neridronate. Eur. Urol..

[78] Pittari G., Costi D., Raballo M., Maulucci L., Baroni M.C., Mangoni M. (2006). Intravenous neridronate for skeletal damage treatment in patients with multiple myeloma. Acta Biomed..

[79] Giannini S., Poci C., Fusaro M., Egan C.G., Marcocci C., Vignali E., Cetani F., Nannipieri F., Loy M., Gambino A. (2021). Effect of neridronate in osteopenic patients after heart, liver or lung transplant: A multicenter, randomized, double-blind, placebo-controlled study. Panminerva Med..

[80] Moretti A., Liguori S., Paoletta M., Gimigliano F., Iolascon G. (2022). Effectiveness of Neridronate in the Management of Bone Loss in Patients with Duchenne Muscular Dystrophy: Results from a Pilot Study. Adv Ther..

[81] Forni G.L., Perrotta S., Giusti A., Quarta G., Pitrolo L., Cappellini M.D., D’Ascola D.G., Pignatti C.B., Rigano P., Filosa A. (2012). Neridronate improves bone mineral density and reduces back pain in β-thalassaemia patients with osteoporosis: Results from a phase 2, randomized, parallel-arm, open-label study. Br. J. Haematol..

[82] Iolascon G., Moretti A. (2019). Pharmacotherapeutic options for complex regional pain syndrome. Expert Opin. Pharmacother..

[83] Resmini G. (2015). Treatment of complex regional pain syndrome. Clin. Cases Miner. Bone Metab..

[84] Varenna M., Adami S., Rossini M., Gatti D., Idolazzi L., Zucchi F., Malavolta N., Sinigaglia L. (2013). Treatment of complex regional pain syndrome type I with neridronate: A randomized, double-blind, placebo-controlled study. Rheumatology.

[85] Varenna M., Braga V., Gatti D., Iolascon G., Frediani B., Zucchi F., Crotti C., Nannipieri F., Rossini M. (2021). Intramuscular neridronate for the treatment of complex regional pain syndrome type 1: A randomized, double-blind, placebo-controlled study. Ther. Adv. Musculoskelet Dis..

[86] Varenna M., Zucchi F., Failoni S., Becciolini A., Berruto M. (2015). Intravenous neridronate in the treatment of acute painful knee osteoarthritis: A randomized controlled study. Rheumatology.

